# Interplay of hippocampal long-term potentiation and long-term depression in enabling memory representations

**DOI:** 10.1098/rstb.2023.0229

**Published:** 2024-07-29

**Authors:** Hardy Hagena, Denise Manahan-Vaughan

**Affiliations:** ^1^ Medical Faculty, Department of Neurophysiology, Ruhr University Bochum, Bochum 44780, Germany

**Keywords:** synaptic plasticity, hippocampus, learning and memory, rodent cognition, LTP, LTD

## Abstract

Hippocampal long-term potentiation (LTP) and long-term depression (LTD) are Hebbian forms of synaptic plasticity that are widely believed to comprise the physiological correlates of associative learning. They comprise a persistent, input-specific increase or decrease, respectively, in synaptic efficacy that, in rodents, can be followed for days and weeks *in vivo*. Persistent (>24 h) LTP and LTD exhibit distinct frequency-dependencies and molecular profiles in the hippocampal subfields. Moreover, causal and genetic studies in behaving rodents indicate that both LTP and LTD fulfil specific and complementary roles in the acquisition and retention of spatial memory. LTP is likely to be responsible for the generation of a record of spatial experience, which may serve as an associative schema that can be re-used to expedite or facilitate subsequent learning. In contrast, LTD may enable modification and dynamic updating of this representation, such that detailed spatial content information is included and the schema is rendered unique and distinguishable from other similar representations. Together, LTP and LTD engage in a dynamic interplay that supports the generation of complex associative memories that are resistant to generalization.

This article is part of a discussion meeting issue ‘Long-term potentiation: 50 years on’.

## 1. Introduction

There is broad consensus that persistent, use-driven and experience-dependent modifications of synaptic strength form the cellular basis of long-term information storage and memory updating by the hippocampus. This derives from an abundance of evidence acquired in recent decades that indicates that associative learning drives long-lasting changes in synaptic efficacy [1–3]. Particularly relevant in this regard are forms of synaptic plasticity that persist over periods of hours or days, thereby putatively allowing the retention of information for prolonged periods and opening up time-windows for the building of associations between past and prospective experiences. This, in turn, may be supported by processes such as metaplasticity [4], synaptic tagging [5] and homeostatic plasticity [6]. Persistent forms of synaptic plasticity are likely to enable the generation of linked neuronal ensembles that support the creation and updating of discriminable associative experience. The primary candidates for this process are two forms of Hebbian, homosynaptic synaptic plasticity that can persist for periods of 24 h and longer, namely hippocampal long-term potentiation (LTP) and long-term depression (LTD). These comprise physiological phenomena that are observed in a laboratory setting that reflect activity-dependent increases or decreases in synaptic efficacy. In this review, the current understanding of the molecular basis and subfield-specific differences of persistent hippocampal LTP and LTD is described. In addition, insights gained from studies of persistent (>24 h) LTP and LTD in the dorsal hippocampus of freely behaving rodents in the presence or absence of learning paradigms shall be considered from the perspective of the delineation of their putative roles and their dynamic interplay in the creation of associative (spatial) memories that last for several hours or days in an experimental setting.

## 2. Temporal phases of hippocampal synaptic plasticity

### (a) When is LTP, LTP?

LTP describes a persistent strengthening of synaptic efficacy that occurs in response to specific patterns of stimulation of afferent inputs [7,8]. The vast majority of studies on hippocampal LTP have been conducted *in vitro*, which, on the one hand, has provided extensive knowledge about the molecular mechanisms that enable this process [9], but, on the other hand, restricts observations of changes in synaptic strength (and their related mechanisms) to periods of maximally hours [10] and typically involves scrutiny for circa (*ca*) 60 min (electronic supplementary material, table S1). Customarily, LTP is therefore described as an increase in synaptic strength that lasts for at least 1 h. However, in behaving animals, hippocampal LTP can be monitored for days [11] and months [12], and induction protocols can discriminate between synaptic potentiation of different durations (electronic supplementary material, table S2). These differences in the timelines of LTP studied *in vivo* and *in vitro* have created difficulties in the definitions of what is truly *long-term* potentiation, as opposed to shorter forms that may comprise separate phenomena (short-term potentiation (STP)) [13], or an interim phase distinguishing LTP that lasts for hours [10] from one that lasts for days and weeks [11,12]. Studies of the molecular basis of persistent (>24 h) LTP, for example, in the cornu ammonis-1 (CA1) region *in vivo* have revealed that it can be disambiguated into temporal components comprising STP that requires activation of *N*-methyl-d-aspartate receptors (NMDAR) and lasts for 30–90 min, depending on the subunit composition of the NMDAR [14]. This early phase of LTP then segues into LTP that lasts for 90–180 min in behaving rodents and requires activation of the metabotropic glutamate receptor, mGlu5 [15]. LTP that lasts for more than 3 h requires brain-derived neurotrophic factor (BDNF) [16], whereas CA1 LTP that lasts for longer than 8 h (referred to as late-LTP) requires both protein translation and transcription [17]. Scrutiny of the NMDAR-dependent component of STP (<90 min) *in vitro* has revealed that it can also be disambiguated into different components: a transient (30 min) form that depends on GluN2D-containing NMDAR [13] and a longer-lasting STP that depends on GluN2A-containing NMDAR and can last for at least 90 min [13,14]. This latter STP can be distinguished from LTP that endures for a similar duration in the CA1 region *in vitro* [18]. Taken together, this suggests that the terminology used to define hippocampal LTP needs to be revisited and made more precise. In the context of this review, LTP will be defined as synaptic potentiation that persists for at least 4 h, whereas *persistent* LTP refers to synaptic potentiation that lasts for at least 24 h.

### (b) Hippocampal LTD also occurs in temporal phases

Scrutiny of hippocampal LTD has revealed that it too can be segregated into temporal components. In the CA1 region, for example, antagonism of NMDAR prevents LTD in anaesthetized rats [19] and radically reduces the magnitude of induced synaptic depression in freely behaving rats, as well as curtailing it to a duration of roughly 30 min [20]. Short-term depression (STD) requires BDNF *in vivo* [21] and LTD in freely behaving rats, which is induced after low-frequency afferent stimulation is limited to a period of *ca* 2 h by antagonism of group 1 mGlu receptors [20]. Furthermore, LTD that lasts for longer than 4 h requires immediate early gene (IEG) expression [22] and protein translation [23].

## 3. The cellular and molecular basis of LTP and LTD: there is no single form of LTP or LTD in the hippocampus, and species differences are also evident

**Figure 1.**
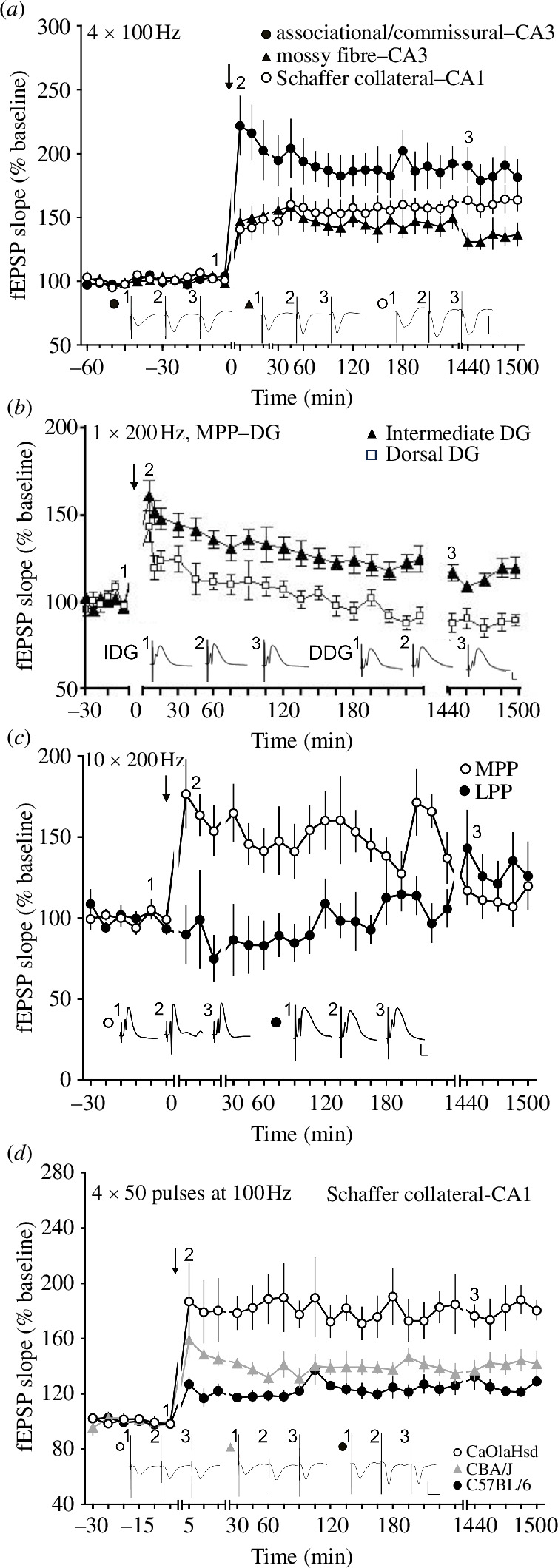
LTP profiles in the cornu ammonis (CA) and dentate gyrus (DG) subfields of the hippocampus of freely behaving young adult rats and mice. (*a*) LTP evoked with high-frequency stimulation (HFS, 100 Hz, four trains of 100 pulses each, 5 min intertrain interval) results in different LTP profiles in associational/commissural (AC)–CA3, mossy fibre (MF)–CA3 and Schaffer collateral (SC)–CA1 synapses of the cornu ammonis of dorsal rat hippocampus. Vertical scale bar: 2 mV, horizontal scale bar: 10 ms. (*b*) Persistent (>24h) LTP can be evoked with a single train of stimuli delivered at 100 Hz in the intermediate DG (closed triangles), but not the dorsal rat DG (open squares). Vertical scale bar: 5 mV, horizontal scale bar: 5 ms. (*c*) Persistent LTP in medial perforant path (MPP) inputs to the dorsal rat DG can be induced with 10 trains of stimuli at 100 Hz and 10 s intertrain intervals. The same stimulation pattern fails to induce LTP at lateral perforant path (LPP)-DG inputs. Vertical scale bar: 4 mV, horizontal scale bar: 3 ms. (*d*) HFS (four trains of 50 pulses each at 100 Hz, 5 min intertrain interval) results in LTP of different magnitudes in CaOlaHsd (open circles), CBA/J (grey triangles) and C57BL/6 (closed circles) mice in SC–CA1 synapses in vivo. To note: C57BL/6 mice develop progressive deafness beginning in the 4th postnatal week; CBA/J mice become blind within 4 weeks after birth; CaOlaHsd mice have no appreciable sensory deficits in their first 12 months of life (see citations below). Vertical scale bar: 2 mV, horizontal scale bar: 10 ms. Black arrows depict the time-points of stimulation. Insets show the analogue examples of field excitatory postsynaptic potentials (fEPSPs) recorded at the time points indicated by the numbers in the graphs. Line breaks indicate change in time scale. Figures modified from [15,24–29].

Although interpretations of the cellular or molecular basis of LTP or LTD in the hippocampus (and their relationship to associative learning) are often merged to reflect a holistic view of hippocampal synaptic plasticity, an examination of the frequency-dependency of LTP in different synaptic subcompartments of the hippocampus reveals very distinct outcomes (figure 1). In this section, focus will be placed on hippocampal synaptic plasticity (>4 h) induced in freely behaving rats and mice, owing to the fact that differences in bath temperature, the composition of perfusion media, the inclusion of excitability suppressants such as picrotoxin in bath media, electrode placement, electrode resistance and slice thickness (to name but a few aspects) make it very difficult to compare outcomes derived *in vitro* [1] (electronic supplementary material, table S1). In general, *in vitro* papers also do not specify whether slices were taken from dorsal, intermediate or ventral hippocampus, which is problematic because not only are the NMDAR complements different in these longitudinal areas of the hippocampus, but LTP in the slice preparation also differs according to the longitudinal axis of the hippocampus [30].

### (a) Frequency-dependency of homosynaptic LTP in hippocampal inputs in freely behaving rats

In freely behaving rats, synaptic potentiation of differing durations can be induced in the dorsal hippocampus by using afferent stimulation frequencies in the range of 100 through 400 Hz (electronic supplementary material, table S2) [31]. Use of frequencies higher than 100 Hz in cornu ammonis synapses will tend to induce epileptiform seizures [31]. Weaker afferent stimulation protocols that induce STP in SC–CA1 synapses induce LTP that lasts for over 4 h at temporoammonic (TA)–CA1 synapses *in vivo* [32] that is NMDAR-dependent [33]. Strikingly, the same 100 Hz protocol that induces LTP at SC–CA1 synapses *in vivo* induces LTP of differing profiles depending on whether mossy fibre (MF)–CA3, commissural–associational (AC)–CA3 or Schaffer collateral (SC)–CA1 synapses are stimulated (figure 1*a*) [11,24]. While MF–CA3 LTP is NMDAR-independent in freely behaving rats [34], it requires activation of NMDAR at AC–CA3 [15] or SC–CA1 synapses [20]. Furthermore, although reciprocal heterosynaptic LTP can be induced in lateral perforant path (LPP) and medial perforant path (MPP) inputs to the CA3 *in vivo* [35], differences in the conditions leading to homosynaptic LTP have been reported in these synapses [36].

To induce persistent LTP in dentate gyrus (DG) synapses, higher afferent stimulation frequencies (typically 200 Hz) are required. At MPP–DG synapses LTP (>24 h) that is NMDAR-dependent can be induced by repetitive stimulation at 200 Hz, whereas LTP (>24 h) that is induced by 400 Hz stimulation is dependent on activation of L-type voltage-gated calcium channels [37]. The frequency-dependency of LTP in DG synapses is not uniform, however: one burst of 200 Hz stimulation induces STP (<2 h) at dorsal MPP–DG synapses, whereas the same afferent patterns induce persistent LTP (>24 h) at intermediate MPP–DG synapses (figure 1*b*) [25]. Furthermore, 10 bursts of 200 Hz stimulation induce persistent LTP at suprapyramidal dorsal MPP–DG synapses, whereas the same afferent pattern delivered to infrapyramidal dorsal MPP–DG synapses fails to induce LTP [38]. The frequency-dependency of synaptic plasticity also differs substantially at dorsal MPP–DG and LPP–DG synapses in freely behaving rats; for example, repeated bursts at 200 Hz result in potent LTP at MPP–DG synapses that lasts for at least 24 h, whereas the same protocol when applied to LPP–DG synapses results in synaptic depression that lasts for about 2 h (figure 1*c*) [39]. Moreover, Abraham & Goddard [40] have reported that MPP–DG and LPP synapses express heterosynaptic forms of synaptic plasticity, such that potentiation in one input is accompanied by depression in the other. In addition, examination of morphological changes of DG synapses after induction of LTP *in vivo* revealed input-specific increases in perforated axospinous synapses in MPP–DG, but not LPP–DG inputs [41] that were accompanied by an input-specific increase in axodendritic synapse density in synapses that expressed heterosynaptic LTD. These findings indicate that all three processes (MPP–LTP, LPP–LTP and heterosynaptic LTD) may be functionally and morphologically distinct. Taken together, these findings highlight that, depending on the synapses/hippocampal subfields involved, LTP in the hippocampus is highly frequency-dependent, which argues against using only one afferent frequency when scrutinizing LTP experimentally and argues for the likelihood that LTP in the different hippocampal synapses is distinct.

### (b) Frequency-dependency of homosynaptic LTP in hippocampal inputs in freely behaving mice

The dorsal CA1 region of freely behaving mice exhibits a very different response profile with regard to LTP induction in compared to rats. In the CA1 region, afferent frequencies that are lower or higher than 100 Hz are ineffective (electronic supplementary material, table S2) [42], whereas it is the impulse number and pattern delivered at 100 Hz (rather than the stimulus frequency) that determine whether STP or LTP is induced [43]. Moreover, theta-burst stimulation is ineffective *in vivo* [43]. At dorsal MPP–DG synapses, 400 Hz stimulation is needed to induce persistent LTP [44]. Here, however, a confound emerges in terms of the interpretation of LTP data obtained from mouse hippocampus: the majority of studies both *in vitro* and *in vivo* have been conducted in C57BL/6 mice that were established as a mouse strain by the Jackson Laboratory in 1948 and have since given rise to well over 220 generations [45]. Drift in the resultant genetic pools has led to the Jackson substrain being referred to as C57BL/6J and the NIH substrain as C57BL/6N, which differ, in turn, from European or Asian variants of these breeding lines [42]. The C57BL/6J and C57BL/6N substrains differ in terms of their responses to sensorimotor and fear conditioning [46,47], as well as spatial memory [48]. Most of the inbred mouse strains used in brain research show differences in their stress sensitivity and anxiety [48–51], brain amine levels [52] and differences in spatial and non-spatial memory [48,53,54]. They also exhibit differences in the distribution of hippocampal afferents and synapses [53]. This makes a general interpretation of the conditions leading to synaptic plasticity in mouse hippocampus and its relevance for associative learning very difficult.

But there is a further confound to using C57BL/6J mice for studies of synaptic plasticity and associated learning behaviour: this mouse strain exhibits presbycusis, whereby it begins losing its auditory acuity at four weeks of age starting with behaviourally relevant high ultrasound frequencies [55]. By five months of age, the mice can only hear frequencies in the range of stress vocalizations, as well as sonic frequencies, and over the course of their adult lifetime become completely deaf [56]. This has consequences for both synaptic plasticity and spatial learning: C57BL/6J mice exhibit significantly reduced LTP both *in vitro* and *in vivo*, as well as impaired spatial memory compared to a mouse strain that exhibits no sensory deficits and a mouse strain that becomes blind shortly after birth [26] (figure 1*d*). C57BL/6J mice also exhibit different social behaviour compared to outbred mice [57] and impaired attention compared to 129SvEv mice [58] that do not display age-related hearing loss [59]. These confounds emphasize the necessity to examine plasticity-related processes and synaptic plasticity in multiple strains and species of rodents, as well as using a variety of afferent stimulation frequencies, in order to better understand their relevance for learning and memory.

### (c) Molecular characteristics of persistent forms of LTP in different hippocampal subfields

The differences in persistent (>24 h) forms of LTP across the hippocampal subfields also become apparent when one compares their molecular dependencies (electronic supplementary material, table S3*a*). Induction of persistent LTP at dorsal SC–CA1, TA–CA1, AC–CA3, MPP–CA3 and MPP–DG synapses typically depends on activation of NMDAR [20,36,37], whereas persistent dorsal MF–CA3 LTP is NMDAR-independent [34] in line with *in vitro* studies that have shown that this form of LTP depends on activation of pre-synaptic kainate receptors *in vitro* [60]. LPP–CA3 LTP is opioid receptor-dependent and not NMDAR-dependent [36]. Although persistent LTP in all of the hippocampal subfields depends on protein synthesis (electronic supplementary material, table S3*a*), one striking difference is evident with regard to MF–CA3 synapses *in vivo*, which also show a requirement of protein synthesis for STP [61]. Furthermore, although LTP at SC–CA1 synapses is arguably predominantly mediated by post-synaptic mechanisms [62–64,7], strong evidence exists that MF–CA3 LTP is induced by pre-synaptic mechanisms [65] and perforant path (PP)–LTP may have both pre- and post-synaptic components depending on whether MPP [37] or LPP inputs [66] are activated. A putative pre-synaptic component to PP–DG LTP has not yet been verified *in vivo*, however.

### (d) Frequency-dependency of homosynaptic LTD in hippocampal inputs in freely behaving rats and mice

**Figure 2.**
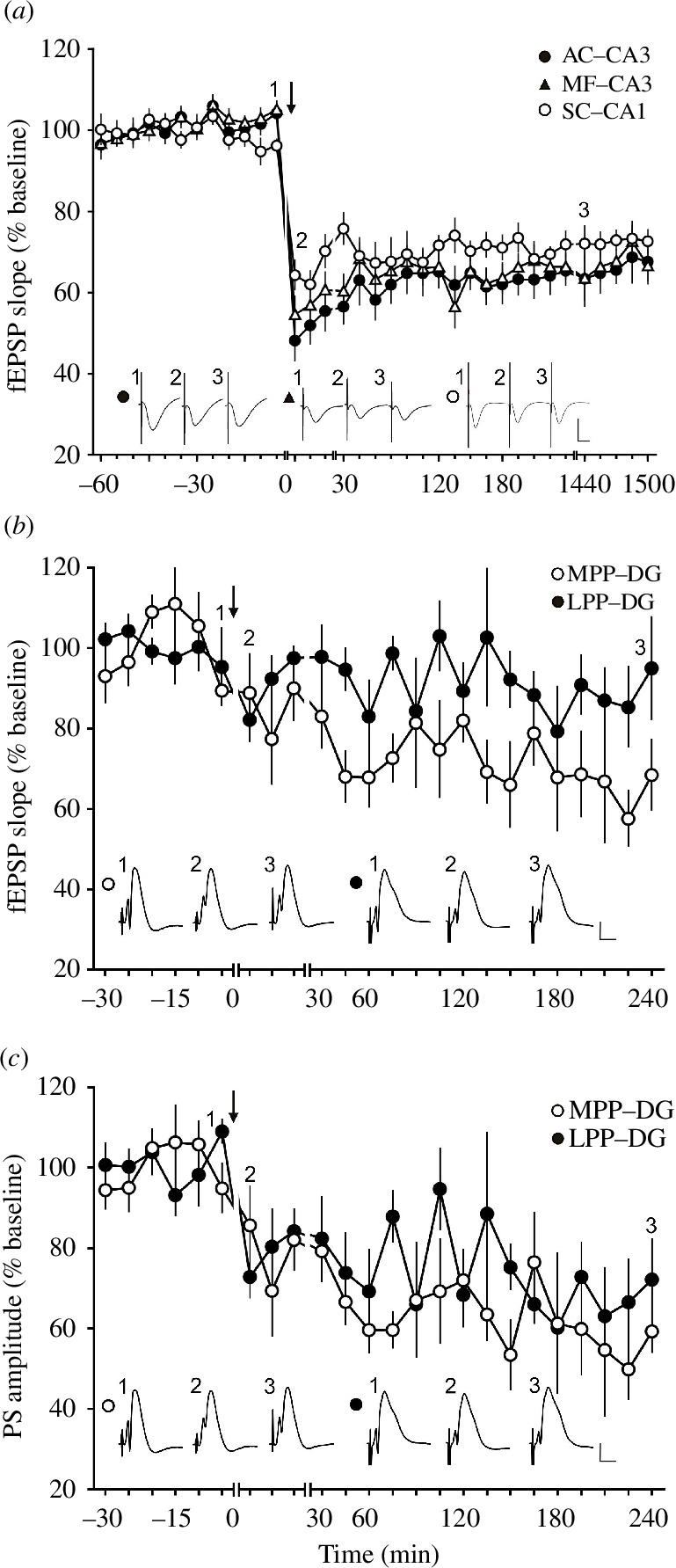
LTD profiles in the cornu ammonis (CA) and dentate gyrus (DG) subfields of the rat hippocampus. (*a*) LTD evoked with low-frequency stimulation (LFS, 900 pulses at 1 Hz) results in different LTD profiles at AC–CA3, MF–CA3 and SC–CA1 synapses in the hippocampus in freely behaving rats. Vertical scale bar: 2 mV, horizontal scale bar: 10 ms. (*b*,c) Result of the same stimulation protocol (900 pulses at 1 Hz) at lateral perforant path (LPP, closed black circles) and medial perforant path (MPP, open circles)–DG synapses. Vertical scale bar: 5 mV, horizontal scale bar: 5 ms. Black arrow depicts the time-point of stimulation. Insets show analogue examples of fEPSPs recorded at the time points indicated by the numbers in the graphs. Line breaks indicate changes in time scale. Figures modified from: (*a*) [69,70]. (*b*,*c*) [39,71].

In contrast to hippocampal LTP in freely behaving rats, the frequencies with which persistent LTD can be induced in hippocampal subfields in rats are quite limited both *in vitro* and *in vivo* (electronic supplementary material, table S4*a*,*c*). In freely behaving rats, regardless of the subfield, 1 Hz afferent stimulation induces persistent (>24 h) input-specific LTD in all dorsal subfields [24,67,68] (figure 2*a–c*), whereby STD can be induced by reducing the pulse number from typically 900 to 600 [24,71] or 300 [72]. Faster frequencies of 3 or 5 Hz induce STD in freely behaving rats [73]. In the murine hippocampal slice preparation, LTD that lasts for up to 2 h can be induced using different variations of 1 Hz stimulation (electronic supplementary material, table S4*b*). In contrast, it is really difficult to induce LTD by afferent stimulation alone in the dorsal hippocampus of freely behaving mice (electronic supplementary material, table S4*d*) [42–44]: different afferent stimulation frequencies (1–10 Hz) and impulse numbers (100–1800) result in STD of differing magnitudes and durations [74], although input-specific NMDAR-dependent LTD can be easily induced in mice by coupling afferent stimulation with spatial learning [75].

### (e) Molecular characteristics of persistent forms of LTD in different hippocampal subfields

Despite the homogeneity of its electrophysiological induction conditions, the molecular dependency of persistent LTD is very different depending on the hippocampal subfield (electronic supplementary material, table S3*b*). Whereas persistent SC–CA1 and AC–CA3 LTD depend on the activation of NMDAR [15,20], persistent LTD at MF–CA3 synapses [61] or MPP–DG synapses [76] are NMDAR-independent. Furthermore, persistent MPP–DG LTD does not require protein synthesis [76], whereas SC–CA1 and AC–CA1 LTD do [23,61]. Additionally, protein synthesis inhibition curtails STD at MF–CA3 synapses *in vivo* [61]. Other differences emerge when the involvement of catecholaminergic receptors in persistent LTD is considered: pharmacological antagonism of β-adrenergic receptors has no effect on LTD that is induced by 1 Hz (900 pulses) stimulation of SC–CA1 synapses in rats [1], but it prevents the maintenance of LTD beyond 4 h at MPP–DG synapses [77]. Pharmacological antagonism of dopamine D1/D5 receptors prevents LTD (>24 h) at SC–CA1 and MPP–DG synapses [1], but has no effect on persistent LTD at MF–CA3 [78] synapses in freely behaving rats.

Taken together, it is clear that there are multiple forms of persistent LTP and persistent LTD in the distinct hippocampal subfields that are characterized by their different frequency-dependencies, expression profiles and molecular dependencies and that are likely to subserve different kinds of information encoding. From an intuitive perspective, this makes sense: the DG is likely to have evolved ahead of the cornu ammonis and may thus fulfil functions that are distinct from those of this structure, such as the acquisition of information about spatial bearing relative to allocentric information [79] and pattern separation [80,81]. All subfields of the hippocampus receive inputs from the entorhinal cortex (EC) [82], theoretically receiving similar information at any given moment in time. However, depending on the synaptic targets ofthe EC within the hippocampal subfields [83], EC afferent terminations within compartments of the respective dendritic trees of the recipient neurons [84], and coupling of EC input with information transferred within the trisynaptic circuit [85,86], this information will be transformed in different ways, resulting in different outcomes in terms of the magnitude, persistency and direction of change of synaptic strength depending on the hippocampal subfield concerned. This interpretation is also consistent with the widespread belief that the different hippocampal subfields fulfil different roles in the storage and recall of associative memories [87].

## 4. Synaptic plasticity is not the only change in synaptic efficacy that occurs in the hippocampus

It would be remiss to fail to mention that synaptic plasticity is not the only physiological process that modulates synaptic strength in the hippocampus. In addition to LTP and LTD, two other cellular phenomena have been described in the dorsal hippocampus that either indirectly or directly instigate changes in synaptic efficacy, namely metaplasticity [88] and slow-onset potentiation (SOP) [89]. Both phenomena exhibit features that are mechanistically distinct from LTP; for example, inhibitory autophosphorylation of calcium calmodulin-dependent kinase II (CAMKII) is required for hippocampal metaplasticity that is induced by patterned afferent stimulation of LPP, but not MPP–DG synapses [90], although LTP typically requires CAMKII. This may relate to neuroregulatory control of metaplasticity by neurogranin [91]. SOP cannot be induced by homosynaptic afferent stimulation parameters [92] but rather occurs in response to activation of G-protein-coupled receptors [89,93,94].

Metaplasticity reflects the property that the prior experience of a synapse has an impact on the characteristics of the synapse’s plasticity response to a subsequent synaptic experience [95]. This can mean, for example, that the same afferent stimulation pattern can result in hippocampal LTP or LTD, depending on the recent past experience of the synapse [1]. Also, the frequency-dependency of synaptic plasticity can shift (to a higher or lower frequency) depending on the prior experience of the synapse [90,96,97]. One striking feature of this phenomenon is that effects occur within a limited time-window after a metaplastic event [98,99]. Moreover, metaplasticity may require synaptic tagging [100] and, thus, could form a substrate for the binding of temporally proximal events into an associative representation. Hippocampal information processing is highly state-dependent, and effects are reflected by changes in the coupling of neuronal oscillations at theta–gamma frequencies [101]. In freely moving rats, identical afferent stimulation frequencies can result in robust LTP, STP, or no change in synaptic efficacy in the absence of any overt change in behaviour, although changes in the coupling of theta- and gamma-frequency neuronal oscillations during the stimulation predict the plasticity outcome [15]. Furthermore, stress changes the thresholds for the induction and maintenance of synaptic plasticity [102–104], as can alterations of the action of neuromodulators in the hippocampus [105]. Thus, metaplasticity is very likely to occur in response to changes in hippocampal homeostasis that can either be experience-dependent or state-dependent, which in turn determines if, and how, recent experience is stored in the form of synaptic plasticity.

SOP describes a gradual potentiation of field potentials that emerges with a latency of several minutes and continues to develop and reach a plateau hours after the initiating event [89,93]. A caveat is that SOP always appears with a delay of several minutes *in vivo* after its initiation [93,106], raising the question as to what the functional relevance of the delayed and incremental increase in excitability could be. LTP, but not SOP, in the CA1 region involves an input-specific increase in synaptic efficacy [92], although the latter may occur in the DG in conjunction with SOP [107]. A striking characteristic of SOP is that it can be induced in anaesthetized rodents [67,106] by afferent stimulation parameters and/or neuromodulatory conditions that induce hippocampal LTD in freely behaving rodents [20,76,77,108], suggesting that it may play a state-dependent role in homeostatic plasticity processes [6]. Thus, the elevation of synaptic excitability enabled by SOP may facilitate the integration and association of information along a prospective timescale by lowering the threshold for the induction of synaptic plasticity for a period of minutes or hours after SOP has been initiated.

## 5. Causal evidence that LTP and LTD support the acquisition and retention of long-term associative experience

Early reports that pointed to a role for LTP in the encoding of associative memory were inferred through experiments conducted in parallel: interventions that prevented hippocampal LTP also impaired spatial learning in separately conducted investigations in rodents (electronic supplementary material, table S5) [109–111], or it was reported, in transgenic animal models, that deficient LTP was associated with deficient spatial memory (electronic supplementary material, table S5) [112–114]. Subsequent studies described the direct relationship of learning with LTP (electronic supplementary material, table S6*a*). For example, recordings from multiple field electrodes placed in the CA1 region of freely behaving rats revealed that a proportion of the recording sites exhibited synaptic potentiation and/or LTP after one-trial foot shock learning [3]. Coupling afferent stimulation of the perforant path afferents with spatial learning events has revealed that cumulative spatial learning [115] and appetitive task-specific learning [116] promote the expression of LTP in the DG. In contrast, learning about novel space facilitates the expression of input-specific hippocampal LTP at MPP–DG, AC–CA3, MF–CA3 and SC–CA1 synapses in freely behaving rats [11,24,71] (electronic supplementary material, table S*6a*). Despite the widespread belief that LTP comprises the cellular basis of learning, ‘proof of principle’ studies of this kind are surprisingly rare, however.

Nonetheless, considering the abovementioned findings that hippocampal LTP may encode one-trial fear memory [3] and that neurons that encode fear memory can generalize easily to integrate other experiences [117], one possibility is that hippocampal LTP may enable the rapid recording of the general schema of an associative experience. This possibility is corroborated by reports that LTP expands the informational content of hippocampal synapses [118] and prompts structural plasticity [119], as well as by findings that hippocampal LTP is facilitated by cumulative spatial learning experience [2] and that temporally spaced inductions of LTP are cumulative and serve to improve spatial learning [120]. Moreover, LTP can be induced by burst depolarizations of one second or less [121,122], suggesting that LTP induction could plausibly occur as soon as an associative experience is perceived as being novel. Reinforcement of the initially acquired LTP may then occur cumulatively: afferent volleys (arguably triggered by increases in attention and arousal driven by the initial LTP-inducing experience) occurring on the backdrop of post-synaptic depolarization (which is mediated by the initial LTP) could then serve to reinforce and prolong LTP [123].

**Figure 3.**
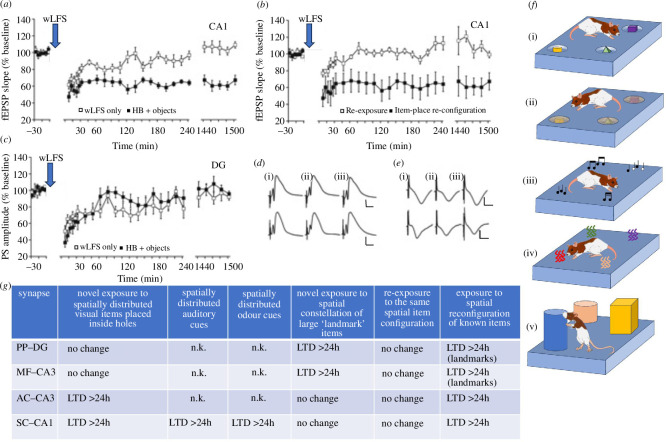
Functional differentiation of the facilitation of LTD by spatial content learning. (*a*) Novel exploration by rats of objects placed in holeboard (HB) holes, during weak low-frequency stimulation (wLFS, 1 Hz, 900 pulses) results in LTD (>24 h) in SC–CA1 synapses. Afferent stimulation with wLFS only results in STD that lasts for *ca* 30 min. Arrows in (*a*–*c)* indicate when wLFS was applied (in the presence or absence of item–place exploration). (*b*) Re-exposure to the same objects in the same holeboard positions in conjunction with wLFS fails to result in LTD. However, exposure of the animals to a spatial re-configuration of the same objects results in LTD (>24 h). (*c*) Stimulation of PP inputs to the DG with wLFS results in STD that persists for <60 min. Novel exposure of rats to objects placed in holeboard holes in conjunction with wLFS fails to induce LTD. (*d*,*e*) Analogue examples of evoked potentials recorded from the DG (*d*) or the CA1 region (*e*) prior to wLFS (i), 5 min post-wLFS (ii) and 24 h after wLFS (iii) in an animal that received wLFS only (top row) and in an animal that received wLFS during the exploration of objects in the holeboard holes (bottom row). (*f*) In SC–CA1 synapses, LTD is expressed when wLFS is applied during novel exploration of novel objects placed in holeboard holes (i) [68], or novel objects concealed under sand inside holeboard holes [11] (ii). LTD is also expressed when wLFS is applied in conjunction with the novel exploration of spatially discriminable auditory frequencies that emanate from loudspeakers placed under holes in the floor (iii) [124] , or spatially distributed odours that diffuse through holes in the floor (iv) [125]. The DG does not respond with a change in synaptic strength to any of these conditions: rather, it expresses LTD when exploration of novel constellations of large landmark features of the environment occurs in conjunction with wLFS (v) [1]. (*g*) Summary of responses of different hippocampal synapses to the abovementioned conditions. PP–DG synapses and MF–CA3 synapses express LTD following exploration of novel configurations of landmark items in space. The AC–CA3 and SC–CA1 synapses do not respond to this kind of information. In contrast, LTD is expressed in these synapses following exploration of novel constellations of items concealed in holeboard holes. PP–DG and MF–CA3 synapses do not respond to subtle item–place information. n.k.: not known. Panels (*a*–*f*) are modified from [11].

In contrast to LTP, where a substantial change in the appearance of the spatial environment is needed to promote LTP that lasts for more than 24 h [1], LTD is facilitated by changes in spatial content, even within a familiar environment (electronic supplementary material, table S6*b*) [2]. The most robust behavioural instigators of hippocampal LTD to be identified thus far are *de novo* item–place experience and the updating (spatial re-configuration) of item–place information. In SC–CA1 synapses of freely behaving rats, persistent LTD is enabled when brief afferent stimulation (which is sufficient to induce transient synaptic depression when applied in the absence of learning) is coupled with novel (or updated) learning of information about spatial constellations of visual [11,68], odour [1] or auditory information [1] (figure 3*a,b,* *f*). For this, physical motion in space is not needed: the presentation of spatial configurations of items on a computer screen is also sufficient to prime for induction of LTD (>24 h) at SC–CA1 synapses [1]. Furthermore, effects are not species-specific: mice also respond with LTD to novel or updated item–place information, whereby in this case only test–pulse stimulation of SC–CA1 synapses in conjunction with novel spatial content learning is needed for LTD to become manifest by this event [75].

Effects are input-specific [1], but differ depending on the hippocampal subfield concerned: while SC–CA1 synapses express LTD in response to learning about spatial constellations of discretely placed cues that can only be discovered if the animal is right beside them, the DG expresses LTD when rats learn about the locations of large, overt items in space [71] (figure 3*a–e*). The CA3 region displays a hybrid function in this regard: MF–CA3 synapses express LTD in response to large overt item–place features, but AC–CA3 synapses behave like SC–CA1 synapses and only express LTD in response to subtly placed items that have to be proximately viewed in order to be localized in space [24] (figure 3). These findings not only indicate that a functional differentiation is evident in hippocampal subfields with regard to the kinds of spatial content that facilitate LTD, but they also show that the hippocampus can use odour and auditory items as a substitute for, or in addition to, visual items to create a record of spatial experience that is supported by persistent (>24 h) LTD.

## 6. Interplay of LTP and LTD enables the acquisition and retention of detailed spatial representations

Fluorescence *in situ* hybridization, used to exploit the property of immediate early genes (IEGs) to exhibit time-dependent peaks in somatic expression that are specifically linked to a behavioural or physiological event [126], has opened up opportunities to examine to what extent the abovementioned forms of learning-related LTP and LTD result in somatic information encoding and/or structural plasticity associated with synapse remodelling [119,127]. While the IEG, *Arc*, exhibits peak somatic expression 5–6 min after a specific experience [128], *Homer1a* exhibits peak somatic expression 30–40 min after a specific event [129]. Immediately after peak somatic expression, the IEG diffuses into the cytoplasm [126], meaning that the detection of somatic IEG expression can serve as an accurate indicator of which neurons engaged in a specific experience-dependent event.

Homer1a is involved in experience-dependent remodelling of the post-synaptic density [130], including increasing both the clustering of α-amino-3-hydroxy-5-methyl-4-isoxazolepropionic acid receptors (AMPAR) [131], as well as the proportion of GluA2-containing AMPAR [132]. In contrast, Arc reduces surface AMPAR expression on synapses [133]. Moreover, Arc accumulates in non-potentiated synapses [134] and contributes to the weakening of synapses by promoting AMPAR endocytosis [135]. To what extent Arc specifically accumulates in synapses that undergo LTD is unclear. The facilitation of LTP by spatial learning results in widespread somatic Homer1a expression across hippocampal subfields [136]. In contrast, learning-facilitation of LTD results in somatic Homer1a expression that is limited to the subfields that express LTD; for example, LTD facilitation by learning about landmark configurations is limited to neurons of the DG and CA3 regions [136]. Examination of somatic IEG expression triggered by exploration of novel items in the holes of a novel holeboard revealed that fusing both novel (LTP-related and LTD-related) events triggers subfield-specific increases in Arc and Homer1a expression [137] that correspond to the subfields in which LTD is expressed [71]. This raises the possibility that LTP and LTD occur in a dynamic partnership: the one (LTP) serving to identify the neural network in which information will be stored and the other (LTD) serving to modify this representation.

**Figure 4.**
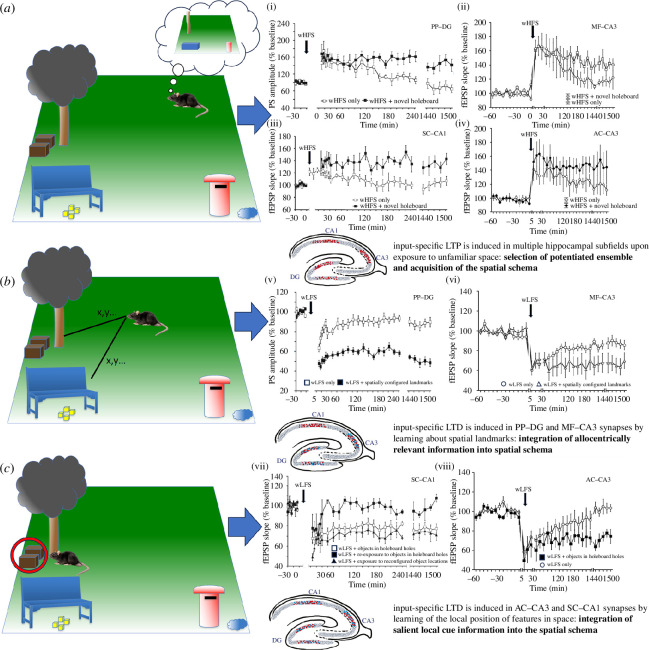
In this concept, a rat is introduced to a completely novel spatial environment where high-contrast items (tree, park bench and letterbox) can be perceived from their initial positions without the need for visual acuity. Movement in the environment allows the animal to acquire metric information about its position relative to these landmark features, as well as to locate salient local features that can only be discovered when the animal is close to them (such as crates that could be used for shelter, food remnants that are under the park bench or water that is beside the letterbox). (*a*) Initial exposure to a novel spatial environment results in immediate induction of LTP in an input-specific manner through hippocampal subfields (red neurons within the top hippocampus schema). By this means a neuronal ensemble is selected in a distributed hippocampal network that serves to retain a schematic representation of the spatial environment. (i–iv) Input-specific LTP (>24 h) is induced in PP–DG (i), MF–CA3 (ii), SC–CA1 (iii) and AC–CA3 (iv) synapses when exposure to entirely novel space is coupled with weak afferent stimulation (weak high-frequency stimulation, wHFS). (*b*) If time is spent moving within the environment, allocentric representations are cumulatively created that permit the acquisition of dimensional, orientational and directional (e.g. landmark) information that allows the integration of allocentrically relevant details into the initially acquired spatial schema. (v-vi) This information is encoded by means of LTD (>24h) in PP–DG (v) and MF–CA3 (vi) synapses (blue neurons within the middle hippocampus schema). (*c*) Salient local details of the environment (i.e. information that can only be found by means of proximal exploration and which is integrated into the allocentric reference frame) are acquired by means of LTD (> 24h) that is expressed in SC–CA1 (vii) and AC–CA3 synapses (viii) (blue neurons within bottom hippocampus schema). By this means the spatial representation is modified and refined, such that it can be discriminated from other similar representations. Graphs are modified from [1,11,24].

This interpretation is supported by data from an electrophysiological study where a novel holeboard containing novel items was presented to rats during test–pulse stimulation of SC–CA1 synapses [68]. What emerged was a potentiation of synaptic responses that segued into synaptic depression. Mechanistically, one could envisage that LTP selects the pan-hippocampal neuronal network that serves as the primary scaffold for information storage and that LTD acts to dynamically enhance the resolution and uniqueness of these potentiated synapses (see, for example, [138]), thereby permitting the storage and disambiguation of similar experiences. When an animal is exposed to a novel environment, LTP is rapidly induced at selected synapses throughout the hippocampus [11,71] (figure 4). If the environment is salient enough or enough time is spent exploring the environment, LTD in PP–DG and MF–CA3 synapses serves to modify the ensemble such that allocentric orientational details are included, whereas LTD at AC–CA3 and SC–CA1 synapses enables the retention of more localized information about the content of the environment [24,71] (figure 4). We propose that this interplay between LTP and LTD is functionally very meaningful: after childhood and arguably young adulthood, most of what we learn is likely to use schemata of past associative experiences [139,140] as the basis for new associative learning. After our first exposure to, e.g. a city park, our next exposure to a park in a different city will use the previous schema to create a new representation that can be disambiguated from the last. We do this over and over in life. It is feasible that LTP serves as the basis for the schema that is re-used for the encoding of similar or updated associative events and that LTD ensures that each generated representation is nonetheless unique.

An interesting possibility is that the sensory features of, e.g. a novel city park result in the recruitment of hippocampal engram cells [141], via LTP, into an ensemble that will repeatedly respond to these cues when the individual is exposed to any generic city park in the future . Depending on the circumstance, this will result in retrieval of the original park memory (pattern completion) or the creation of a new city park memory (pattern separation, driving modifications of the representation). The site of access of the original schema is likely to be the hippocampus (CA1, CA3, DG subfields) [142] even if remote memory of the schema is stored in the neocortex [143], whereas the updating of the schema to integrate new information recruits information processing in both the hippocampus and cortical regions such as the retrosplenial cortex [142,144].

With the caveat in mind that the experiments were done in experience-naive animals, it was shown that LTP can be maintained for months in the hippocampus of rats [145], thus raising the possibility that the scaffold of the schema may be retained in the hippocampus for prolonged periods and reactivated by appropriate sensory input from the EC. Here, the possibility exists that LTD not only serves to modify the schema to enable the creation of a new representation that is distinguishable from other similar memories, but also that it plays a role in driving changes in neocortical representations: induction of LTD in the CA1 region in association with spatial content learning drives somatic IEG expression in the retrosplenial cortex [146].

## 7. How is the hippocampus instructed to express either persistent LTP or persistent LTD?

Although the frequency-dependency of electrophysiologically induced persistent LTP and LTD largely determines the direction of change in synaptic strength (electronic supplementary material, tables S1–S2), this is not the case for endogenously induced synaptic plasticity. Either STD or LTD can be induced by test–pulse stimulation of hippocampal afferents in conjunction with spatial content learning [68,75]. This process can even curtail LTP [75]. Afferent stimulation that emulates theta frequency oscillations in the hippocampus (which typically occur during spatial exploration: [147,148]) can either promote or interfere with LTP [149,150]. While stimulation on the peak of theta induces LTP, stimulation on its trough induces LTD [151,152]. This raises the question as to the means by which the hippocampus interprets incoming signals such that information encoding in the form of LTP or LTD occurs.

As mentioned earlier, hippocampal information processing is state-dependent. Elevations in attention trigger increased medial septal release of acetylcholine and glutamate in the hippocampus [153], which change network excitability, drive theta oscillations [154,155] and lower the threshold of LTP induction [96]. Action of septal acetylcholine in the hippocampus also permits that stimuli, that are otherwise subthreshold for induction of synaptic plasticity to successfully induce LTP [154] and supports the generation of spatial representations [156]. The ventral tegmental area (VTA) is a midbrain structure that plays an important role in the perception and binding of reward and punishment-related stimuli [157]. It has been proposed to engage in a feedback loop with the hippocampus that enhances novelty-related firing of cells in both structures [158]. VTA activity may modulate both hippocampal LTP [159] and hippocampus-dependent associative memories [160]. Furthermore, activation of midbrain inputs to the hippocampus fosters the persistence of spatial memory and leads to reactivation of neuronal ensembles that were recruited during spatial learning [161]. These observations suggest that septal and VTA inputs to the hippocampus may facilitate LTP induction during increased arousal related to associative experience.

The locus coeruleus (LC) is a strong candidate for endogenous promotion of hippocampal LTD. Test–pulse stimulation of hippocampal afferents in conjunction with electrophysiological activation of the LC results in input-specific hippocampal LTD that is NMDAR-dependent [105,108,162]. Stimulation of the LC also improves episodic-like memory in rats [108], facilitates spatial memory retention [106] and supports spatial contextual memory updating [163]. The timing of LC activity relative to the induction of LTP *in vivo* can either have no effect on, or depotentiate, recently induced LTP [164]. LC-mediated hippocampal LTD can be induced by a variety of LC frequencies [162] and is evident in both PP–DG and SC–CA1 synapses of freely behaving rats [108], suggesting that this is a very robust phenomenon. Taken together, these findings could suggest that changes in LC firing that are driven by saliency or novelty could enable hippocampal LTD and related encoding of spatial content. The coincidence of informational inputs from other sources is essential; however, LC stimulation fails to induce LTD in freely behaving rats in the absence of test–pulse stimulation of hippocampal afferents [108] and in anaesthetized rodents, in which excitatory responses are consequently depressed [165,166] and the thresholds for induction of synaptic plasticity are increased [167], LC stimulation can induce either SOP [106] or inhibit LTP [163].

## 8. Conclusion

Causal evidence is accumulating that persistent forms of LTP and LTD enable the acquisition and retention of associative memories. While LTP enables the acquisition of the associative schema and initial spatial representations, LTD appears to support the refinement and optimization of the representation such that allocentric and subtle spatial content details are included in the representation [71]. LTD may also enable dynamic updating and adaptive flexibility of engram ensembles [138], thereby ensuring that similar representations can be disambiguated and remain unique. Through this dynamic interplay of hippocampal LTP and LTD, associative representations can be linked and updated, thereby allowing the creation and retention of reliable records of complex experience.

## Data Availability

Supplementary material is available online [168].
